# Optical-Quality Assessment of a Miniaturized Intraocular Telescope

**DOI:** 10.3390/jcm12103375

**Published:** 2023-05-10

**Authors:** Irene Nepita, Raffaele Raimondi, Simonluca Piazza, Alberto Diaspro, Faustino Vidal-Aroca, Salvatore Surdo, Mario R. Romano

**Affiliations:** 1 Nanoscopy, Istituto Italiano di Tecnologia, Via E. Melen 83, 16152 Genova, Italy; 2 Genoa Instruments s.r.l., Via E. Melen 83, 16152 Genoa, Italy; 3 Department of Biomedical Sciences, Humanitas University, 20090 Milano, Italy; 4 DIFILAB, Department of Physics, University of Genoa, 16146 Genoa, Italy; 5 Department of Scientific Affairs, Medevise Consulting, 67000 Strasbourg, France; 6 Dipartimento di Ingegneria dell’Informazione, Università di Pisa, 56122 Pisa, Italy; 7 Eye Center, Humanitas Gavazzeni-Castelli, 24128 Bergamo, Italy

**Keywords:** end-stage age-related macular degeneration, visual impairment, visual prosthesis, implantable ophthalmic device, intraocular lens, optical performance, geometrical aberrations, SING IMT™

## Abstract

Age-related macular degeneration (AMD) causes severe vision impairments, including blindness. An option to improve vision in AMD patients is through intraocular lenses and optics. Among others, implantable miniaturized telescopes, which direct light to healthy lateral regions of the retina, can be highly effective in improving vision in AMD patients. Yet, the quality of the restored vision might be sensitive to the optical transmission and aberrations of the telescope. To shed light on these points, we studied the in vitro optical performance of an implantable miniaturized telescope, namely, the SING IMT™ (Samsara Vision Ltd., Far Hills, NJ, USA) designed to improve vision in patients affected by late-stage AMD. Specifically, we measured the optical transmission in the spectral range 350–750 nm of the implantable telescope with a fiber-optic spectrometer. Wavefront aberrations were studied by measuring the wavefront of a laser beam after passing through the telescope and expanding the measured wavefront into a Zernike polynomial basis. Wavefront concavity indicated that the SING IMT™ behaves as a diverging lens with a focal length of −111 mm. The device exhibited even optical transmission in the whole visible spectrum and effective curvature suitable for retinal images magnification with negligible geometrical aberrations. Optical spectrometry and in vitro wavefront analysis provide evidence supporting the feasibility of miniaturized telescopes as high-quality optical elements and a favorable option for AMD visual impairment treatments.

## 1. Introduction

Age-related macular degeneration (AMD) is the leading cause of blindness in millions of people worldwide [1,2]. Briefly, AMD strikes the center of the retina, namely, the macula, leading to blurry and poorly resolved vision. So far, there is no effective cure for the advanced stage of AMD. Standard treatments for the exudative form of AMD are based on vascular endothelial growth factors (VEGF), with the drawback of requiring frequent intravitreal injections, which not only hamper patients’ compliance to the therapy, but they are also poorly effective—they only slow down the progression of the disease [3]. Recently, the FDA approved the usage of pegcetacoplan for the treatment of advanced geographic atrophy (GA) secondary to AMD, being the only available therapy for this condition. Interestingly, this drug acts on the complement pathway. However, real-world outcomes are still lacking. Genomic tailored therapies are an interesting possible solution, though, not clinically feasible yet [4].

There is also an increasing interest in the development of nanotechnology-based scaffolds or cellular suspensions, which potentially allow regenerating the damaged retinal layers. Despite the active research, there are no commercially available options, and most trials are still in the early phases [5]. As a result, patients affected by advanced AMD are forced to use supportive measures for hypo vision such as external magnifiers or telescopes. Surgical options, on the other hand, are very limited and include macular translocation surgery and, more recently, retinal-pigmented epithelium transplant [5]. In this scenario, the development of effective intraocular implantable optics for late-stage AMD patients may fulfill a considerable unmet need. At present, a wide range of intraocular implants is adopted for patients affected by AMD, such as Lentis Max, IOL-VIP, EYEmax Mono, Scharioth lens or IMT™. These lenses can be compared based on their main features; however, it still difficult to make a realistic comparison in terms of efficacy or safety in the absence of direct comparative studies, in particular, related to parameters such as endothelial cell loss, stages of the indicated AMD, optical design and number of cases studied [6].

Recently, a new intraocular optical device, namely, the SING IMT™ (Samsara Vision Ltd.), has been indicated for improving vision in late-stage AMD patients. The SING IMT™ comprises two optical microlenses, one with positive power (converging lens) and another with a negative power (diverging lens). The two lenses are placed at a relative distance that leads to a miniaturized Galilean telescope with a magnification of 2.7 ± 10% when combined with the human cornea. The entire system is sealed to avoid liquid infiltration during in vivo operations [7]. Once inside the eye, this implant allows light to reach healthier retinal regions, which surround the degenerated macula because only the center of the retina is damaged in AMD disorders. The implant, indeed, enables retinal cells surrounding the macula to image the object.

Although Galilean telescopes have been proposed for visual impairment in AMD treatment [8], the SING IMT™ provides a new eye-implantable solution because it integrates foldable haptics and makes use of a preloaded delivery system, which enables surgery with reduced incision size [7,9,10,11]. As such, the SING IMT™ attempts to combine the pros of rigid IOLs, which are robust against deformations and thus geometrical aberrations, with the benefits of foldable polymeric IOLs, which reduce the incision size and enable minimal invasive surgery. The design of the SING IMT™ provides several improvements with respect to its precursor model IMT™ including reduced surgical complexity and a more standardized implantation procedure. Indeed, the preloaded delivery system and the foldable haptics, significantly reduce the corneal incision from 12 mm to 6–8 mm and generally simplify the surgical procedure with respect to the IMT™. However, overall, the surgery still remains challenging and requires time-consuming steps with respect to the implantation of an acrylic IOL.

The aim of the present study is the in vitro optical quality assessment of the SING IMT™ device. To this end, we measured the geometrical aberrations and the spectral transmittance of the SING IMT™ optical system. Furthermore, we used a second mono-focal implantable lens (Monofocal IOL, +20 Diopters, Model SY60WF, Alcon Laboratories Inc., Fort Worth, TX, USA), widely used in cataract surgery, as a benchmark for the optical quality of the SING IMT™ device. We found that the SING IMT™ has excellent optical transmission in the visible with negligible geometrical aberrations, which make this intraocular device a valuable tool for vision-improving applications.

## 2. Materials and Methods

### 2.1. Transmission Spectra Measurement Setup

**Figure 1:**
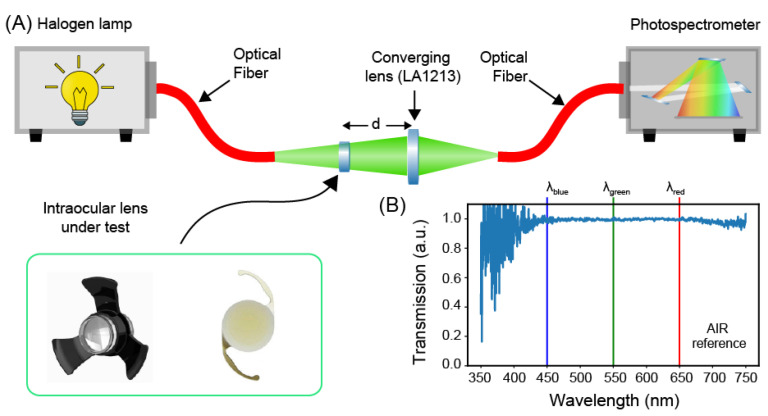
Optical transmission spectroscopy. (**A**) Schematic description of the optical fiber setup used for measuring the transmission spectra of the intraocular optics. (**B**) Reference spectrum of an ideal transmitter measured in air without any intraocular optical elements in the optical path.

Measurements of the transmission spectra of the intraocular lenses were carried out by means of an optical fiber setup (Figure 1A) [12,13]. In particular, a wideband (350–750 nm) radiation of a tungsten halogen lamp (6 V/30 W, OSRAM) was shed against the element under test through a multimode optical fiber. The lenses under the test were mounted into specific sample holders (Figures S1 and S2) and inserted into the optical path. The light passing through the optics under test was launched into a multimode optical fiber (core diameter of 50 µm and numerical aperture NA of 0.22) by means of a converging lens (LA1213, Thorlabs, Newton, NJ, USA) placed at distance of 50 mm from the IOL, and finally recorded, with a spectral resolution of 0.38 nm, through a photospectrometer (USB200+, OceanOptics, Orlando, FL, USA). 

The setup was designed to ensure that the numerical aperture and focus spot size of the system comprising the converging lens (i.e., LA1213) and the IOLs under test are smaller than the numerical aperture and core diameter of the collecting fiber for all the wavelengths in the interval 350–750 nm (see Supplementary Material: Section S2 and Figure S4). This condition ensures efficient coupling between the transmitted light and the fiber and prevents undesired cut-off wavelengths due to the measuring setup. Possible chromatic aberrations could still cause a minimal axial shifting of the focus, which results in optical losses. To mitigate this problem, we adjusted the relative distance between the optical fiber and the lenses with the aim of maximizing the intensity of light transmitted throughout the entire spectrum. To increase the signal-to-noise ratio of our measurements, the recorded spectrum was averaged over 10 acquisitions, each integrated over a time interval of 50 ms.

A calibration procedure was performed to compensate for the spectral signature of the lamp and to remove possible background contribute. To this end, we first subtracted the dark spectrum, namely, the transmission spectrum with the lamp off, from the recorded spectrum and then we normalized the latter to the spectrum of an ideal transmitter, namely, air (Figure 1B). As such, all the subsequent transmission spectra were measured as variations caused by the element under test with respect to air. Finally, each spectrum was normalized to its maximum, thus neglecting optical losses due to the residual coupling losses between the transmitted light and the collecting optical fiber. 

The following analytical parameters were extracted from the measured transmission spectra: (i) the transmission bandwidth defined as the full width at half-maximum (FWHM) of the transmission spectra; (ii) the band flatness computed as the standard deviation of the transmission values within the FWHM; (iii) the extinction ratios of the blue radiation, by definition the ratio between the transmission at the wavelength of 450 nm and the transmissions at 550 and 650 nm, respectively.

### 2.2. Geometrical Aberrations Measurement Setup

Several methods exist to evaluate the geometrical aberrations of a lens. Examples include measuring the modulation transfer function (MTF) [14,15] or wavefront sensing [16,17] and then extracting from these measurements the wave functions for each aberration. In general, these functions have complicated mathematical formulations for the MTF, especially for high order aberrations, while simple polynomials (i.e., the Zernike base) suffice to reconstruct the recorded wavefront. As such, we selected direct wavefront sensing and Zernike expansion to study the aberrations (up to the 14th order) of the IOLs under test, an approach widely used in ophthalmology and optical metrology [16,17,18,19]. To this end, we designed and assembled a custom setup based on free-propagation of a laser beam and comprising two main functionalities: (i) preconditioning of the laser beam, and (ii) wavefront sensing with a Shack–Hartmann sensor. 

**Figure 2:**
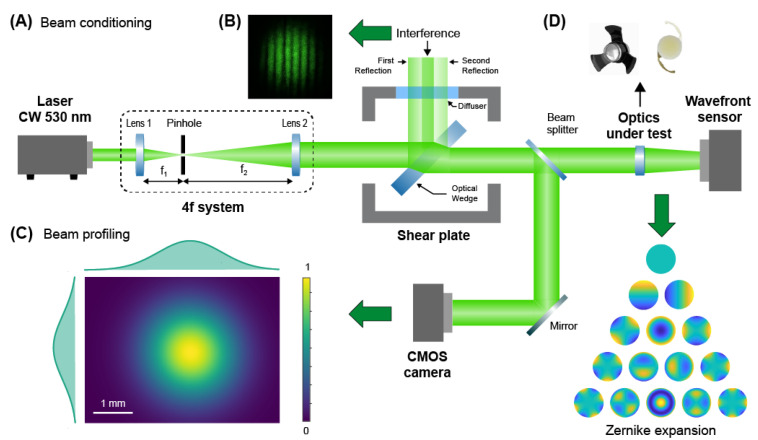
Wavefront sensing setup. (**A**) The beam exiting a laser module is filtered and expanded with two lenses and a pinhole. (**B**) A shear plate is used to set the distance between the lenses, thus ensuring collimation. (**C**) A fraction of the laser beam is imaged and processed with a beam profiling algorithm in order to determine the beam waist. (**D**) The reaming fraction of the beam is directed towards the intraocular lens under test and the resulting wavefront measured and expanded in Zernike polynomials.

The beam conditioning setup (Figure 2A–C) was designed and arranged in order to obtain a laser beam with the following properties: Gaussian shape, collimation, and a beam waist filling the optical aperture of the intraocular device. To this end, a continuous-wave (CW) laser emitting at 530 nm (Coherent, Sapphire, Santa Clara, CA, USA) was expanded by inserting two lenses placed at 2f distance one from the other (with a magnification of 2×), and spatially filtered through a 50-μm pinhole (Figure 2A). The collimation of the laser beam was verified with a shear plate (SI050, Thorlabs) (Figure 2B). A fraction of the beam was redirected into a CMOS camera (DCC1545M, Thorlabs) to compute its size through beam profiling methods (Figure 2C). The beam waist at 4-sigma was 3.2 mm and was in agreement with the clear aperture of the SING IMT™ device. For the measurements with the monofocal IOL, which has an optical aperture of 6.5 mm, an additional afocal system with a 2×-magnification was used to further expand the laser beam. The remaining fraction of the beam was directed towards a Shack–Hartmann sensor (WFS150-5C, Thorlabs) to compute the wavefront distortions and the Zernike polynomials (Figure 2D) [20]. In order to measure the distortions of the wavefront immediately after the IOL under test, the sensor was positioned immediately (a few mm) after the IOL and optically conjugated to the plane where the IOL is for the experiments with the SING IMT™ and the SY60WF, respectively. Moreover, for only measuring the wavefront distortion due to the optical element under test, a calibration was necessary. Specifically, we recorded the wavefront of the preconditioned laser beam only and used it as a reference (Figure S2A). The reference wavefront was substantially flat (Figure S2B) with no evident optical aberrations (Figure S2C). Once calibrated, the optical setup allowed us precisely measuring the wavefront distortions due to any optical elements inserted in the light path. To ensure the reliability of our measurements, each wavefront was computed by averaging 10 acquisitions and 3 wavefronts were recorded for each lens. Moreover, in order to provide high resolution to our measurements, namely, accurate detection of a large number of Zernike terms, we selected a pupil diameter (i.e., 3.3 mm) matching the laser beam waist. For experiments with the SY60WF monofocal IOL, we fulfilled this requirement by inserting a 4f-system with a magnification of 0.5× between the intraocular lens and wavefront sensor. The contribution of these additional optics to the wavefront was subtracted during calibration. All the wavefront sensing experiments were conducted in air as surrounding medium for the optics under test.

## 3. Results

### 3.1. Transmission Properties

**Figure 3:**
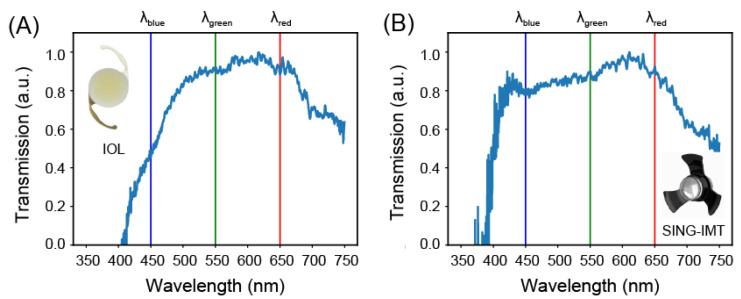
Transmission properties in the UV-Vis. Experimental normalized transmission spectrum of the SY60WF monofocal IOL (**A**) and SING IMT™ device (**B**). The vertical blue, green, and red lines mark the wavelength values of 450, 550, 650 nm.

Figure 3 shows the normalized transmission spectra of the SY60WF and SING IMT™ optics. We found that the SY60WF attenuates the intensity of light in the blue band of the visible spectrum, where the normalized transmission is 0.5 at 𝜆 = 450 nm (Figure 3A). The SING IMT™ device, on the other hand, evenly transmits the entire visible light, attenuates the blue band (transmission 0.8 at 𝜆 = 450 nm), and entirely suppresses the UV components (Figure 3B). 

In order to quantify these effects, we extracted several analytical parameters from the recorded spectra, precisely, both width and flatness of the transmission band along with the extinction ratio between transmitted light intensity at the wavelengths of 450, 550, and 650 nm. Results of this analysis are presented in Table 1. Standard deviation values of the extinction coefficients were calculated measuring the level of noise of our measurements (0.08, 0.13, and 0.12 for air, SY60WF, and SING IMT™, respectively) and applying propagation error to the analytical expression of the coefficients [21]. The same parameters are reported for the case of the ideal transmitter, namely, air. 

We found that both devices substantially exhibit a flat response within their bandwidth. However, the bandwidth of the SY60WF monofocal IOL is 56 nm narrower than that of the SING IMT™. The calculated extinction ratios confirmed the different spectral behavior of the two devices. Indeed, the SY60WF monofocal IOL shows an attenuation of the blue light, with respect to the green and red, of approximately 50%. The SING IMT™, on the other hand, has almost near-unity extinction ratios and no filtering capabilities in the blue spectrum.

### 3.2. Geometrical Aberrations

**Figure 4:**
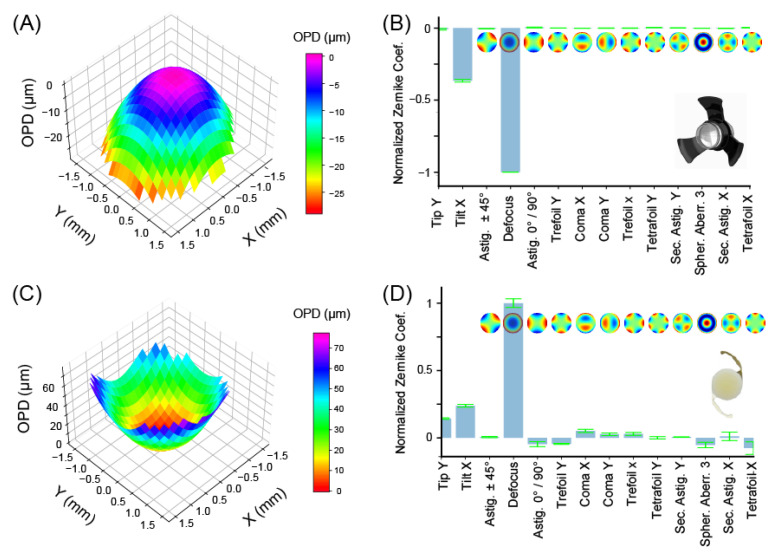
Wavefront sensing and geometrical aberrations characterization. (**A**) Reconstructed wavefront and (**B**) Zernike coefficients of SING IMT™ device. (**C**) Reconstructed wavefront and (**D**) Zernike coefficients of SY60WF monofocal IOL. For clarity of comparison, the Zernike coefficients of each lens are normalized with the absolute value of the defocus term. The optical path difference (OPD) is defined as the difference between the aberrated and the ideal wavefronts. Pupil diameter: 3.3 mm.

Figure 4A shows the wavefront of the SING IMT™ reconstructed as the linear combination of Zernike polynomials. A clear curvature of the wavefront is evident. Specifically, the wavefront concavity indicated that the SING IMT™ effectively behaves as a diverging lens with a focal length of −111.15 ± 0.04 mm in air (see Supplementary Material: Section S3). Considering that once implanted into the eye the aqueous solution cannot infiltrate the SING IMT™, we expect that this system will continue to be diverging in vivo, though, its focal length will be longer—approximately 30%—because of the increased refractive index of the surrounding medium, namely, aqueous humor rather than air. 

The expansion of the wavefront into a Zernike basis allowed us to quantify the extent of the first 14 aberrations. As shown in Figure 4B and Table 2, the SING IMT™ has negligible coma, trefoil, astigmatism, and spherical aberrations, which clearly proves its suitability as optical imaging element. In the same in vitro conditions, the SY60WF monofocal IOL behaves as a converging lens with a focal length of 47.72 ± 0.63 mm, which is in agreement with the specifications and the clinical purpose of this intraocular lens, namely, replacing the crystalline in cataract surgery. As shown in Table 2 and Figure 4D, the Zernike coefficients of the SY60WF are slightly larger with respect to those obtained with the SING IMT™ device, which might be ascribed to possible mechanical stress that the flexible haptics transfer to the lens in the in vitro conditions of our experiments. The larger variability of the Zernike coefficients (std values in Table 2) of the SY60WF with respect to that of the SING IMT^TM^ corroborates the idea that foldable IOLs are more sensitive to the environmental conditions where they operate, at least under in vitro conditions. Although the in vivo scenario might be more favorable for the SY60WF—the liquid environment in the human eye might be a more comfortable place for foldable IOLs—the SING IMT™ resulted less sensitive or totally immune to deformation-induced geometrical aberrations because of its mechanical rigidity. 

## 4. Discussion

Intraocular vision-improving devices, such as implantable lenses, have been recently used in patients affected by various forms of AMD [6]. However, AMD-affected patients usually have poor functional results after cataract surgery with standard monofocal IOL implants [22]. To overcome this problem, novel and more advanced intraocular devices, including miniaturized telescopes, have been developed [23,24,25]. For instance, the IMT™ (Vision Care, Israel) is a monocular implant approved by the US Food and Drug Administration (FDA) and Canadian and European authorities for patients aged at least 55 years with stable and severe vision impairment (BCDVA 20/80 to 20/800). The IMT™ was found effective in improving the visual acuity of patients affected by bilateral central scotomas and due to end-stage AMD, as confirmed by a 2-year study with more than 200 patients [8,26]. Following these results, the IMT™ was further developed, leading to the genesis of the SING IMT™ (Samsara Vision Ltd.), which is the subject of our study and approved by the European authorities. A recent non-comparative retrospective study proves that the optical features of the SING IMT™ system provide acuity improvement in more than 20 eyes [27].

The operational principle of the SING IMT™ is quite simple. Briefly, the SING IMT™ is an ultra-precision wide-angle micro-optic whose magnification compensates for missing scarred macular receptors, thus attenuating the visual impact of scotoma. Specifically, the SING IMT™ in vivo, namely, after replacing the crystalline and once coupled with the cornea, projects an enlarged image into the retina, thus improving visual acuity in AMD patients, though with a reduced visual field of 20° [28]. As such, healthy and lateral portions of the retina sense light, thus improving the vision of patients affected by late-stage AMD. Though proven functional, the quality of the vision recovered with SING IMT™ might be sensitive to its optical features. Open questions include, does the SING IMT™ transmit evenly the visible wavelengths, or some of them are suppressed? Does the SING IMT™ induce geometrical aberrations, which might hamper the imaging process? To answer these questions, we assessed the optical performance of the SING IMT™ under in vitro experimental conditions. In particular, we studied the shape of its transmission spectrum in the UV-visible and measured its geometrical aberrations. A commercial intraocular lens, namely, the SY60WF monofocal IOL, Alcon (branded as Clareon IOL), was used as a benchmark for this study.

The SING IMT™ lens exhibited a quite flat transmission spectrum over the visible interval (i.e., 400–750 nm). Furthermore, the SING IMT™ device and SY60WF monofocal IOL feature high UV-rejection and blue-rejection capabilities, respectively. These results agree with the optical properties of the structural materials of the two optical elements, namely, polymers for the SY60WF monofocal IOL [29,30,31] and fused silica for the SING IMT™ [32,33,34]. Notably, the cut-off wavelength of 400 nm for the SING IMT™, where silica still has a moderate extinction coefficient of 2.7 × 10^−3^, is compatible with the design of the Galilean telescope, which uses two lenses whose optical absorption cumulates as light travels through the device. Moreover, the flatness of the transmission spectrum of the SING IMT™ within its bandwidth, suggests the absence of interferences between light waves reflected at each internal air/silica interface.

The wavefront’s concavity indicated that the SING IMT™ effectively behaves as a diverging lens with a focal length ~2.6-fold higher than that of the crystalline lens [35], which is in agreement with the magnification expected once implanted into the eye, namely, 2.7 ± 10% as by design. Moreover, wavefront sensing and Zernike analysis revealed that both devices are suitable as intraocular imaging elements. In particular, the SING IMT™ showed excellent optical quality with almost zero coma, astigmatism, trefoil, and spherical aberrations. The terms that contribute more to the wavefront shape are tilt-X and defocus. However, it should be noted that defocus and tilt are not real aberrations, since they only shift the focal plane. In other words, a perfect wavefront altered by tilt or defocus still results in an aberration-free image. Notably, this result can be ascribed to the design of the SING IMT™, where the use of flexible polymers is limited to the haptics. The latter are indeed the only elements that could introduce tilting of the wavefront because the optical element is a rigid compound system made of silica microlenses. As such, this design prevents mechanical deformation of the optics [36,37,38,39] and, thus, geometrical aberration, which is a key aspect for high-quality imaging. 

It is essential to highlight that, despite the design improvement in the SING IMT™, some limitations still exist. Indeed, a corneal incision of 6–8 mm raises the surgical complexity of this procedure compared to a standard cataract procedure. Moreover, post-operative astigmatism and endothelial cell loss could represent a concern. Additionally, the effective lens position (ELP) is difficult to estimate due to the thickness of the telescope. Considering these points, future in vivo studies are needed to assess real-life optical outcomes. 

Importantly, although the SING IMT™ has high optical quality, it is still crucial to follow strict patient eligibility criteria to obtain a functional result [26,27]. In particular, patients should be advised of the complete loss of stereopsis that results from the lens implant, which is typically executed only in one eye, so that the other eye can be still used to see in the surrounding space while the implanted eye is fitted for near vision. For this reason, after surgery, six to eight rehabilitation sessions are highly recommended to avoid diplopia and allow proper use of the implanted optical device, regardless of its high optical quality.

## 5. Conclusions

In this work, we assessed the optical quality of the intraocular miniaturized compound lens system, namely, the SING IMT™ device (Samsara Vision Ltd.), through an in vitro characterization of its geometrical aberrations and optical transmittance in the UV-Vis spectrum. We found that the SING IMT™ effectively operates as a diverging lens with homogenous optical transmittance in the visible along with UV-rejection. Moreover, the SING IMT™ can magnify the retinal images, thus making such implantable optics a valuable choice in advanced-stage AMD patients undergoing cataract surgery [22]. Yet, the SING IMT™ exhibited a regular wavefront as confirmed by Zernike’s analysis, which resulted in negligible geometrical aberrations. The feasibility of the SING IMT™ as an aberration-free intraocular implant is evident if compared with intraocular technologies entirely based on flexible polymers, at least under in vitro experimental conditions. We can anticipate that the assessment of the quality of the SING IMT™ still requires studies. In addition to in vivo studies, the extent of peripheral aberrations vs. size of the visual field, which the SING IMT™ provides, is a key aspect to investigate for optimal designs—the wider the field of view, the lower the number of reached healthy cones and the more relevant off-axis aberrations become. 

The selection of an implantable optical element, among standard polymeric IOLs and the more recent Galilean one, is still up to the surgeons and depends on several aspects, including the need of blue-light blocking, the invasiveness of the surgery, or more in general, the patient pathology [40]. However, as we know more about the interaction of light with the retina epithelium and novel miniaturized optics will be developed [41], more effective and minimally invasive IOL will continue to emerge.

## Figures and Tables

**Table 1:** Main features of the recorded transmission spectra for an ideal transmitter (i.e., air), the SING IMT™ optics, and the SY60WF monofocal IOL, respectively.

	Bandwidth (Inf-Sup) (nm)	Flatness (rms)	Extinction Ratio (Blue to Green)	Extinction Ratio (Blue to Red)
AIR	350–750	0.08	0.98 ± 0.11	0.98 ± 0.11
SY60WF IOL	453.41–749.75	0.13	0.45 ± 0.17	0.45 ± 0.17
SING IMT™	397.08–749.75	0.12	0.94 ± 0.25	0.91 ± 0.25

**Table 2:** Absolute values of Zernike coefficients of the SING IMT™ and SY60WF monofocal IOL. Pupil diameter: 3.3 mm. Please note that, since the optics under test have different clinical/functional purpose and design, in order to obtain a reliable comparison, the Zernike coefficients in Figure 4B,D, were normalized to their defocus term.

Aberrations	SING-IMT (µm)		SY60WF-IOL (µm)	
	Mean	Std	Mean	Std
Tip Y	−0.057	0.014	2.727	0.031
Tip X	−2.652	0.020	4.529	0.071
Astigmatism ± 45°	−0.034	0.002	0.115	0.026
Defocus	−7.247	0.002	18.988	0.206
Astigmatism 0°/90°	−0.028	0.007	−0.878	0.119
Trefoil Y	0.005	0.002	−0.849	0.028
Coma X	−0.007	0.002	0.992	0.073
Coma Y	−0.004	0.004	0.514	0.065
Trefoil X	−0.009	0.004	0.559	0.069
Tetrafoil Y	0.025	0.001	−0.005	0.056
Sec. Astig. Y	0.003	0.004	0.115	0.014
Spher. Aberr. 3	−0.011	0.001	−1.005	0.130
Sec. Astig. X	−0.016	0.002	0.213	0.200
Tetrafoil X	0.017	0.004	−1.435	0.295

## Data Availability

All the obtained data used to support the findings of this study are available from the corresponding author upon reasonable request.

## Supplementary Materials

The following supporting information can be downloaded at: https://www.mdpi.com/article/10.3390/jcm12103375/s1, Section S1: Sample holders engineering; Figure S1: SING IMT™ device holder; Figure S2: Monofocal intraocular lens (SY60WF, Alcon) device holder; Figure S3: Calibration of the wavefront sensing setup; Section S2: Design of the optical spectroscopy setup; Figure S4: Effective numerical aperture and focus size of the compound system SY60WF + LA1213 and SING IMT™ + LA1213 lenses; Section S3: Focal length and defocus relationship.
