# Supplementary material for: Optical-Quality Assessment of a Miniaturized Intraocular Telescope
Source: J Clin Med. 2023 May 10;12(10):3375. doi: 10.3390/jcm12103375 (PMC10219409; doi:10.3390/jcm12103375)
Supplement: Supplementary file 1 [file jcm-12-03375-s001.zip › jcm-2308226-supplementary.pdf]

Journal of
Clinical Medicine

MDPI

Article

Optical-Quality Assessment of a Miniaturized Intraocular Telescope

Irene Nepita [1,2], Raffaele Raimondi [3], Simonluca Piazza [1,2], Alberto Diaspro [1,4], Faustino Vidal-Aroca [5], Salvatore Surdo [2,6,*] and Mario R. Romano [3,7]

1 Nanoscopy, Istituto Italiano di Tecnologia, Via E. Melen 83, 16152 Genova, Italy
2 Genoa Instruments s.r.l., Via E. Melen 83, 16152 Genoa, Italy
3 Department of Biomedical Sciences, Humanitas University, 20090 Milano, Italy
4 DIFILAB, Department of Physics, University of Genoa, 16146 Genoa, Italy
5 Department of Scientific Affairs, Medevise Consulting, 67000 Strasbourg, France
6 Dipartimento di Ingegneria dell'Informazione, Università di Pisa, 56122 Pisa, Italy
7 Eye Center, Humanitas Gavazzeni-Castelli, 24128 Bergamo, Italy
* Correspondence: salvatore.surdo@ing.unipi.it; Tel.: +39-050-2217-504

Keywords: end-stage age-related macular degeneration; visual impairment; visual prosthesis; implantable ophthalmic device; intraocular lens; optical performance; geometrical aberrations; SING IMT™

Section S1. Sample Holders Engineering

In order to guarantee high precision in the alignment of the optical lens being tested by the probing light, we designed and prepared custom mounts and cage systems that take into account the overall dimensions and geometries of the intraocular devices. For the SING IMT™ device, an adjustable circular iris diaphragm (SM05D5D, Thorlabs) was mounted into a 30 mm cage plate with removable sample holder (CFH1R, Thorlabs) (Figure S1). The implantable device was positioned in the iris aperture and the three haptic wings were blocked with a rubber O-ring, avoiding additional forces and, thus, limiting possible off-axis misalignments.

We could not use this sample holder for the monofocal intraocular lens (SY60WF, Alcon) because of its elasticity, which might cause undesired deformations under the action of uneven mechanical pressure. For this reason, a sample holder compatible with standard mounting systems of 1-inch optics (Figure S2) was designed and 3D-printed.

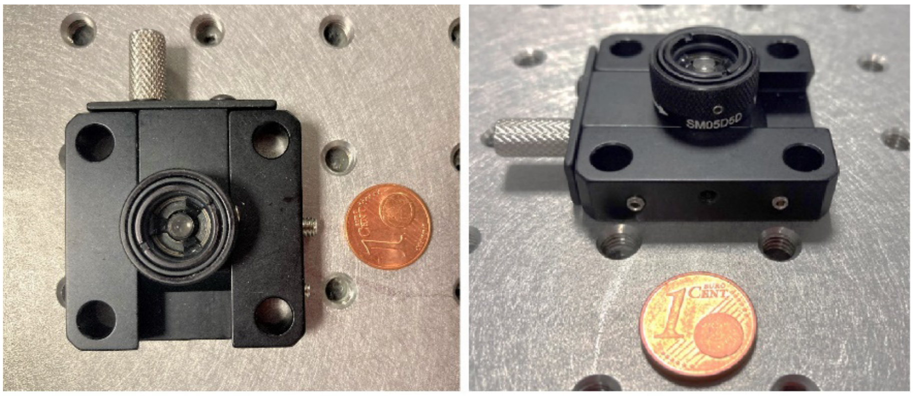

Figure S1. SING IMT™ device holder. Pictures showing the sample holder mounting the SING IMT™ and used to precisely insert the optical element under test into the measurement setup.

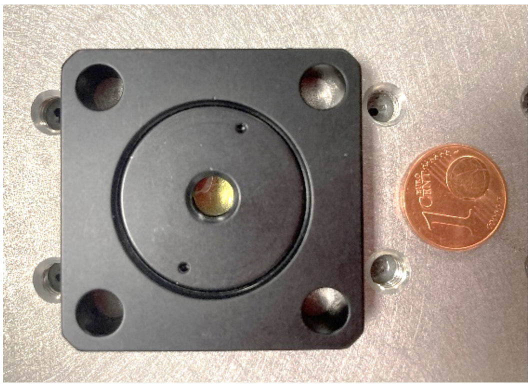

Figure S2. Monofocal intraocular lens (SY60WF, Alcon) device holder. Picture of the 3D-printed sample holder for the IOL lens.

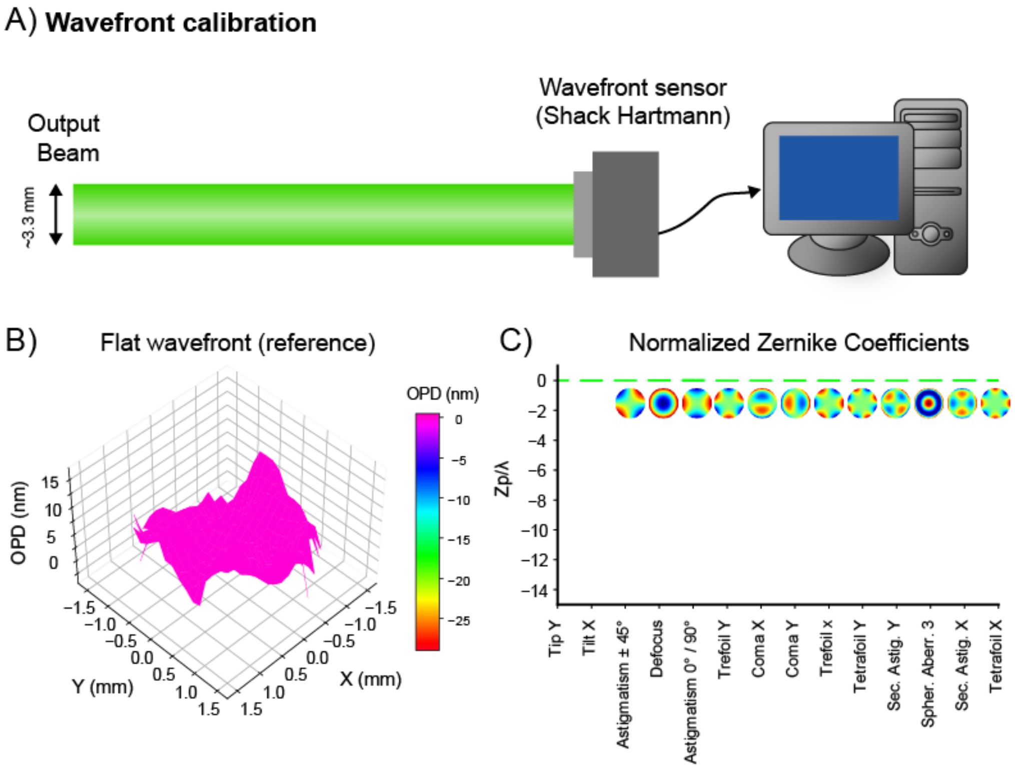

Figure S3. Calibration of the wavefront sensing setup. A) Schematic description of the optical setup used to calibrate the wavefront sensor. B) Measured reference wavefront and corresponding geometrical aberrations C). The optical path difference (OPD) is defined as the difference between the aberrated and the ideal unaberrated wavefronts.

Section S2. Design of the Optical Spectroscopy Setup

In order to couple light into the readout multimode optical fiber, the system made of the IOL under test and the converging lens (LA1213) must fulfill two conditions: 1) the diameter of the light spot at the focal plane (fiber entrance) of the system must be smaller than the core diameter of the fiber (W = 50 μm); and 2) the numerical aperture of the system must be smaller than that of the fiber (NA = 0.22). Simply put, the acceptance angle of the fiber must be larger than the cone of light to be coupled. Moreover, conditions 1) and 2) must be satisfied for all the wavelengths of this study.

The effective focal length (f) of the compound system made of the LA1213 lens and IOLs under test can be computed as:

$$\frac{1}{f} = \frac{1}{f_1} + \frac{1}{f_2} - \frac{d}{f_1 f_2} \quad \text{(S1)}$$

Copyright: © 2023 by the authors. Licensee MDPI, Basel, Switzerland. This article is an open access article distributed under the terms and conditions of the Creative Commons Attribution (CC BY) license (https://creativecommons.org/licenses/by/4.0/).

where f_1 and f_2 are the focal lengths of the IOL and the LA1213 lens, respectively, and d = 50 mm their distance; in our case, $f_1 \approx$ 47 mm and $f_1 \approx$ -110 mm for the SY60WF and the SING IMT at the wavelength of 530 nm, and f_2 = 50 mm. From eq. S1, it is simple to verify that both systems have an effective focal length of ~ 50 mm. The effective numerical aperture (NA) of the system can be approximated as:

$$NA = \frac{D}{2f} \quad \text{(S2)}$$

with D being the optical aperture of the system, which in our case is limited by the optical aperture of the IOL; D = 3.2 mm for the SING IMT and D = 6.5 mm for the SY60WF, leading to NA = 0.065 and 0.032 for the SY60WF+LA1213 and SING IMT+LA1213, respectively.

Note that the NA = 0.22 of the optical fiber is significantly larger than that of both tested optics (**condition 1**). Considering a Gaussian light intensity distribution and diffraction-limited system, we can compute the focus size (W) with the Airy formula $W = 1.22\ \lambda/NA$, which in our case leads to W~9.5 µm for the SY60WF+LA1213 and W~20 µm for the SING IMT+LA1213 at λ = 530 nm. Please note that in both cases the focus spot size is smaller than the core diameter (50 µm) of our fiber (**condition 2**).

Let us now consider the effect of possible chromatic aberrations on the effective numerical aperture and spot size. As shown in Figure S4, the variation of the lenses' refractive index with the wavelength of light results in an effective numerical aperture and focus spot size that fulfill **conditions 1 and 2** for **all the wavelengths** in the interval 400-750 nm.

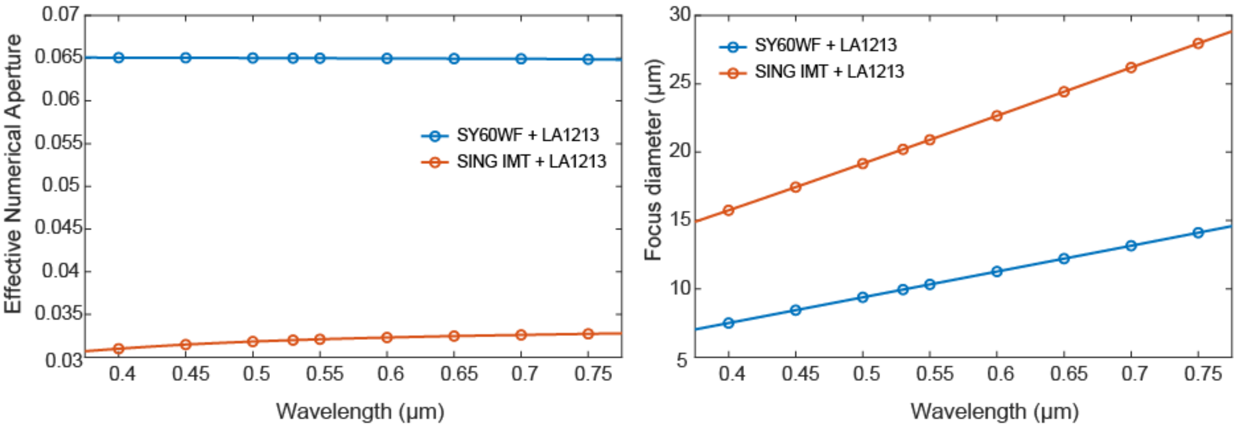

Figure S4. Effective numerical aperture and focus size of the compound system SY60WF+LA1213 lenses (blue line and symbols) and SING IMT+LA1213 lenses (red line and symbols), respectively. In all instances, the effective numerical aperture and focus size are smaller than the numerical aperture (0.22) and core diameter (50 µm) of the optical fiber guiding the transmitted light to the photospectrometer.

Notably, the dispersion of the refractive index also leads to minor changes of the effective focal length of the system. Similarly, possible deviations from the thin lens condition shift the focus along the optical axis. To mitigate this problem, we moved the optical fiber along the optical axis to maximize the transmitted light over the entire spectrum. This procedure, along with the largely satisfied conditions for efficient photon coupling into the readout optical fiber, is robust against undesired light filtering. As a matter of fact,

the reference spectrum of the ideal transmitter, namely air (Fig. 1B), is almost flat and does not show any cut-off wavelengths.

Section S3. Focal Length and Defocus Relationship

The defocus polynomial can be used to compute the transfer function of the effective measured lenses as follows:

$$I(x,y) = exp[-jk(n_L - 1)WF(x,y)] \quad \text{(S3)}$$

where k is the wavenumber, n_L the lens refractive index, and $WF(x,y)$ the absolute wavefront given by the defocus polynomial, i.e., $WF(x,y) = Z_3\sqrt{3}\left[\frac{8}{d^2}(x^2+y^2) - 1\right]$, with Z_3 being the defocus coefficient and d the pupil diameter of our setup. We further find that the transfer function can be rewritten as:

$$I(x,y) = exp\left[jk(n_L - 1)Z_3\sqrt{3}\right]exp\left[-j\frac{k}{2}(n_L - 1)16Z_3\sqrt{3}(x^2+y^2)\right] \quad \text{(S4)}$$

We can neglect the first exponential contribution in eq. S4 since the absolute phase is not important to describe the phase transformation the lens operates. By comparison with the transfer function of a standard lens, namely $I(x,y) = exp\left[-j\frac{k}{2f}(x^2+y^2)\right]$, we find that the optical power (i.e., the inverse of the focal length) of the effective lens with wavefront curvature $WF(x,y)$ can be calculated, solving the identity of:

$$\frac{k}{2}(n_L - 1)16Z_3\sqrt{3}(x^2+y^2) = \frac{k}{2f}(x^2+y^2) \quad \text{(S5)}$$

which, after further calculation, results in:

$$\frac{1}{f} = (n_L - 1)\frac{16Z_3\sqrt{3}}{d^2} \quad \text{(S6)}$$

and, hence:

$$f = \frac{d^2}{16Z_3\sqrt{3}}\frac{1}{(n_L-1)} \quad \text{(S7)}$$

Considering the effective lens made of silica (n_L=1.4878) and polymer (n_L= 1.4337) for the SING IMT and SY60WF, the effective focal length of the two lenses are -111.15 ± 0.04 mm and 47.72 ± 0.63 mm, respectively.

Disclaimer/Publisher's Note: The statements, opinions and data contained in all publications are solely those of the individual author(s) and contributor(s) and not of MDPI and/or the editor(s). MDPI and/or the editor(s) disclaim responsibility for any injury to people or property resulting from any ideas, methods, instructions or products referred to in the content.
